# Correction to: Bioactive glass nanoparticles induce strong preferential cytotoxicity and excessive ROS‑mediated oxidative stress and apoptotic genomic DNA damage in non‑small lung cancer cells

**DOI:** 10.1007/s00210-026-05007-3

**Published:** 2026-01-24

**Authors:** Hanan R. H. Mohamed, Amira H. Yehia

**Affiliations:** Department of Zoology, Faculty of Science, Cairo University, Giza, Egypt

**Correction to: Naunyn-Schmiedeberg's Archives of Pharmacology**

10.1007/s00210-025-04636-4

The original version of this article unfortunately contained a mistake.

The author noticed that Table 4 contains an error that requires correction to ensure accuracy and clarity of the results. The specific issue is as follows: incorrect names of the reported genes.

The correct Table 4 is as follows:

**Table 4:** Expression level of apoptotic (p53 and Bax) and anti-apoptotic Bcl2 genes in in untreated control and A549 lung cancer cells treated with doxorubicin (2 µM) or varying concentrations of BGNPs (2.89, 5.78, and 11.56 µg/ml) for 48 h

Treatment	Concentration	Fold change in the expression level of		
		p53 gene	Bax gene	Bcl2 gene
Untreated cells	0.00	1.00 ± 0.00 ^a^	1.00 ± 0.00 ^a^	1.00 ± 0.00 ^a^
Doxorubicin	2µM/ml	2.59 ± 0.04 ^b^	3.75 ± 0.01 ^b^	0.67 ± 0.02 ^b^
Y_2_O_3_NPs	1/4 IC50 (2.89 µg/ml)	4.39 ± 0.01 ^c^	5.25 ± 0.04 ^c^	0.42 ± 0.03 ^b^
	1/2 IC50 (5.78 µg/ml)	7.55 ± 0.04 ^d^	9.23 ± 0.04 ^d^	0.28 ± 0.02 ^c^
	IC50 (11.56 µg/ml)	9.32 ± 0.03 ^e^	12.20 ± 0.03 ^e^	0.08 ± 0.01 ^d^
ANOVA		*F*=45528.73 *p*<0.001	*F*=72364.37 *p*<0.001	*F*=893.69 *p*<0.001

Results are expressed as mean ± SD. Results were analyzed using One Way Analysis of Variance (ANOVA) followed by Duncan's test. Means with different superscript letters indicate statistical significant difference among the compared untreated control and treated A549 lung cancer cells at *p*<0.001

The corrections do not affect the interpretation of the data or the study conclusions, as no data were altered; only the names of the tested genes were corrected.

The original article has been corrected.

